# Neutrophilic epitheliotropism, proposed as an auto‐inflammatory condition of neutrophilic urticarial dermatosis including Schnitzler syndrome, is also observed in Japanese cases

**DOI:** 10.1111/1346-8138.17067

**Published:** 2023-12-06

**Authors:** Hitomi Nakaizumi, Naotomo Kambe, Hiroyuki Irie, Yo Kaku, Masakazu Fujimoto, Hajime Yoshifuji, Yasuhiro Kazuma, Kazumoto Katagiri, Takuro Kanekura, Kenji Kabashima

**Affiliations:** ^1^ Department of Dermatology Kyoto University Graduate School of Medicine Kyoto Japan; ^2^ Center for Allergy Kyoto University Hospital Kyoto Japan; ^3^ Department of Diagnostic Pathology Kyoto University Hospital Kyoto Japan; ^4^ Department of Rheumatology and Clinical Immunology Kyoto University Graduate School of Medicine Kyoto Japan; ^5^ Department of Hematology and Oncology Kyoto University Graduate School of Medicine Kyoto Japan; ^6^ Department of Dermatology Dokkyo Medical University Saitama Medical Center Koshigaya Japan; ^7^ Department of Dermatology Kagoshima University Graduate School of Medical and Dental Sciences Kagoshima Japan

**Keywords:** neutrophil, neutrophilic epitheliotropism, neutrophilic urticarial dermatosis (NUD), Schnitzler syndrome (SchS), skin biopsy

## Abstract

Schnitzler syndrome (SchS) is a rare autoinflammatory disease characterized by bone pain, recurrent fever, leukocytosis, and elevated C‐reactive protein, along with an urticaria‐like rash and monoclonal immunoglobulin (Ig)M or IgG gammopathy. Notably, the condition is distinguished by a relatively persistent recurrent urticarial‐like rash. Histopathological features observed in the skin comprise diffuse neutrophil infiltration into the dermis, absence of dermal edema, and vascular wall degeneration, all of which classify SchS as a neutrophilic urticarial dermatosis (NUD). Accumulated histological data from skin biopsies of patients with NUD have revealed a sensitive histopathological marker for NUD, acknowledged as neutrophilic epitheliotropism, which has been proposed as reflecting an autoinflammatory condition. In this report, we present three SchS patients: two men (ages 55 and 68) and a woman (age 75), all displaying neutrophilic epitheliotropism in their skin biopsy specimens. Additionally, a review of eight previously reported SchS cases in Japan identified neutrophilic epithliotropism in five cases. These findings suggest that the inclination of neutrophils toward the epithelial tissue could aid in confirming diagnoses of NUD in most cases that need to be differentiated from conventional urticaria. Consequently, we emphasize that acknowledging neutrophilic epithelial predilection as a hallmark of NUD is critical for expediting early diagnosis and appropriate treatment for SchS.

## 1 INTRODUCTION

Schnitzler syndrome (SchS) is considered to be an acquired form of an autoinflammatory syndrome due to its late onset, typically around 50 years of age,^1^ and its resemblance to clinical phenotypes of cryopyrin‐associated periodic syndrome (CAPS).^2^ The diagnosis of SchS is based on the Strasbourg criteria established by Lipsker et al.,^3^ which includes chronic urticarial rash and monoclonal immunoglobulin (Ig)M or IgG as two obligate criteria. Minor criteria include a neutrophilic dermal infiltrate on skin biopsy, recurrent fever, objective findings of abnormal bone remodeling with or without bone pain, and leukocytosis or elevated C‐reactive protein (CRP). As mentioned in the minor criteria, SchS may present a different pathological picture from ordinary urticaria.

Kieffer et al.^4^ claim that some autoinflammatory diseases, including SchS, present a pathological picture known as neutrophilic urticarial dermatosis (NUD). Clinically, patients with NUD have a recurrent or chronic cutaneous eruption consisting of macules, papules, or plaques, with individual lesions resolving within 48 h and may or may not be pruritic. Pathologically, a skin biopsy specimen shows a diffuse neutrophilic infiltrate in the dermis with interstitial involvement, but without vessel wall alteration (especially parietal necrosis) and dermal edema.

Broekaert et al.^5^ further examined NUD pathology by analyzing patients with SchS, CAPS, Still disease, systemic lupus erythematosus (LE), Sjögren syndrome, and other conditions. Comparison of these samples with skin biopsies of neutrophilic urticaria, neutrophil‐expressing LE, and eruptive drug reactions that did not meet NUD criteria showed neutrophilic infiltration also in the epidermis, hair follicles, sebaceous glands, sweat glands, and ductal epithelium in NUD. They termed this feature “neutrophilic epitheliotropism”, proposed as an indicator of an underlying autoinflammatory condition. In this report, we reviewed skin biopsies from three experienced SchS cases and found neutrophilic epitheliotropism in all biopsies. Additionally, a review of eight previously reported SchS cases in Japan identified neutrophilic epitheliotropism in five cases. These findings suggest neutrophilic epitheliotropism is a useful feature of NUD and a valuable finding in diagnosing SchS.

## 2 CASE REPORTS

### 2.1 Case 1

**FIGURE 1:**
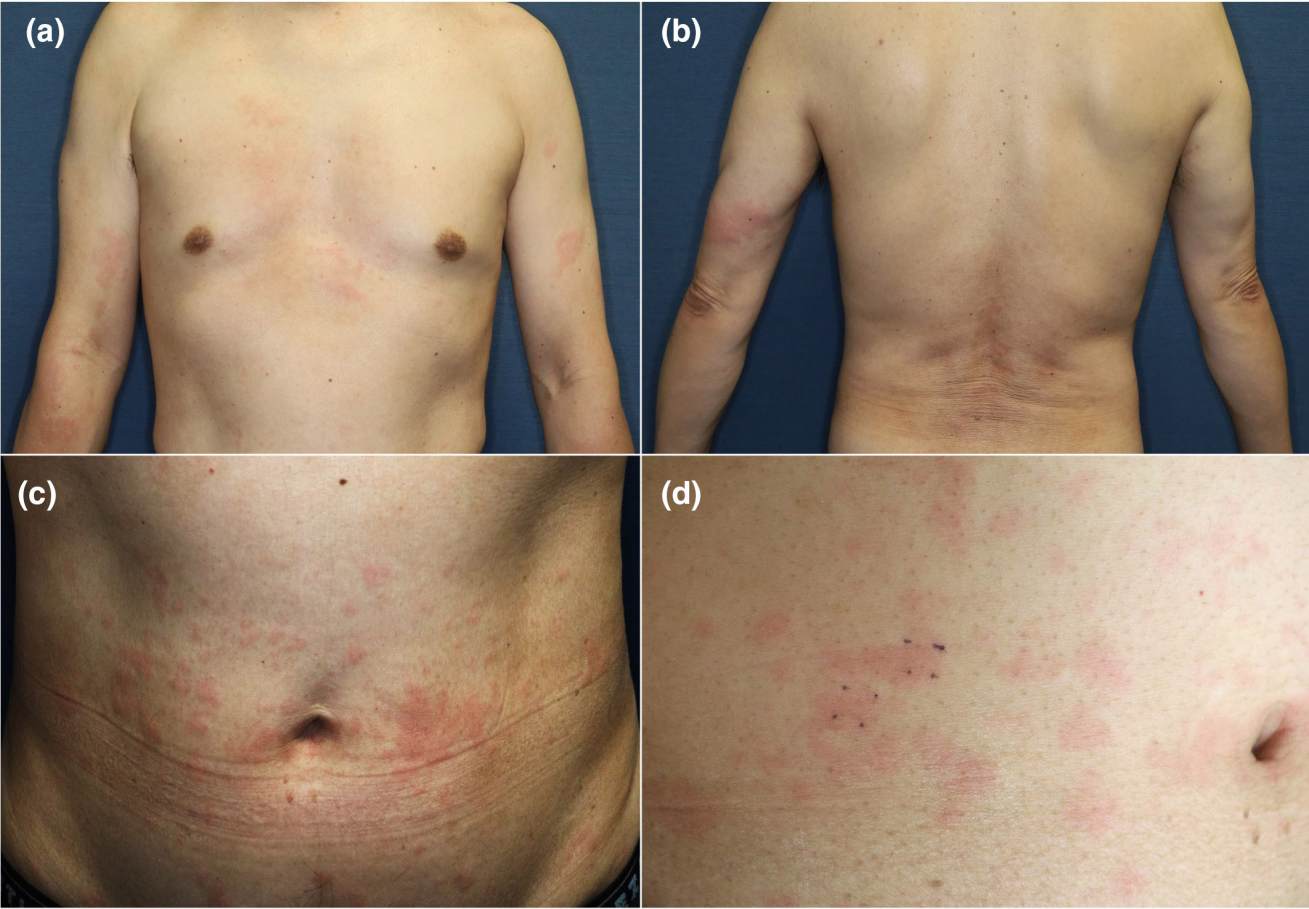
(a–c) Urticarial rash of Case 1. (d) The skin lesion biopsied in Case 1.

**FIGURE 2:**
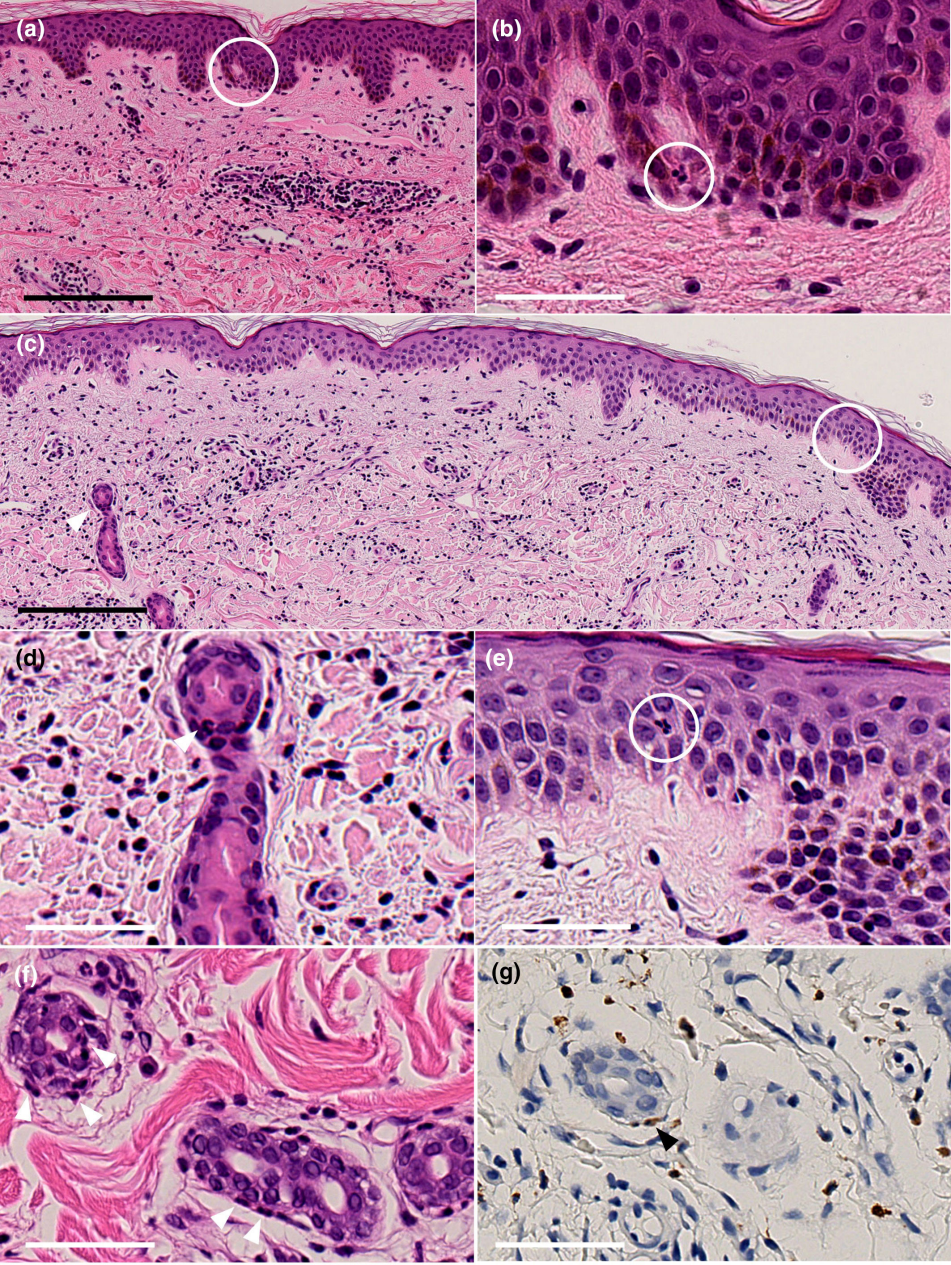
Histopathological findings of the skin lesion. (a) Hematoxylin and eosin (HE) staining of the biopsy from Case 1 (scale bar = 200 μm);white circle indicates neutrophilic epitheliotropism. (b) Higher resolution of the circled area shows a neutrophil in the epidermis observed in Case 1 (scale bar = 50 μm). (c) HE staining of the biopsy from Case 2 (scale bar = 200 μm). (d) The white arrowhead demonstrates a neutrophil within the ductal epithelia of the eccrine gland noted in Case 2 (scale bar = 50 μm). (e) The white circle indicates a neutrophil within the epidermis in Case 2 (scale bar = 50 μm). (f) HE staining and (g) immunohistochemical staining for myeloperoxidase of the biopsy from Case 3. Arrowheads demonstrate neutrophils within the secretory epithelia of eccrine glands and harbored neutrophils around the eccrine glands (scale bar = 50 μm).

A 55‐year‐old man presented to a dermatology clinic with fever, fatigue, and a non‐itching skin rash. The patient had a prolonged fever of 38°C, lasting for a few days, accompanied by nonpruritic urticaria‐like eruptions. Colchicine (1.5 mg/day) was started for suspected SchS, which led to an improvement in his symptoms. However, 7 years after the onset of symptoms, the fever and rash worsened, prompting the patient to seek further examination and treatment at our department. An urticarial skin rash was observed on his chest, abdomen, lower back, and arms (Figure 1). Laboratory examination revealed monoclonal IgM gammopathy (742 mg/dL), neutrophilic leukocytosis (white blood cells [WBC]), 10 580/μL; neutrophils, 70.1%), and slightly elevated CRP (0.74 mg/dL). This case corresponds to Case 27 in our previous report of clinically diagnosed cases in Japan.^1^ A biopsy was performed from the right lower abdomen. Histopathology showed diffuse neutrophilic infiltration within the dermis, as well as neutrophilic infiltration into the epidermis, which could be indicative of NUD (Figure 2a,b).

### 2.2 Case 2

A 68‐year‐old man is reported as Case 2 in a previous study on the efficacy of molecularly targeted drugs for SchS,^6^ and Case 22 in our case summary.^1^ He was undergoing treated with oral prednisolone (5 mg/day) and tocilizumab, experiencing occasional fatigue and fever over 38°C about 1–2 times a week. Laboratory findings showed monoclonal IgM gammopathy (328 mg/dL), neutrophilic leukocytosis (WBC, 13 820/μL; neutrophils, 88.5%), but normal CRP due to tocilizumab. During his first visit to our department, a skin biopsy was performed on his left hypochondrium. Histopathology revealed neutrophilic infiltration into the epidermis and epithelia of eccrine glands, in addition to the dermis (Figure 2c–e).

### 2.3 Case 3

A 75‐year‐old woman is reported as Case 1 in our previous publication^6^ and Case 23 in the case summary.^1^ Additionally, we have reported elsewhere that the skin biopsy tissue of this case showed characteristic inducible skin‐associated lymphoid tissue.^7^ Approximately 3 years after initial diagnosis, tocilizumab was switched to anakinra, an interleukin (IL)‐1 inhibitor, and the skin rash and clinical symptoms improved dramatically, except for monoclonal IgM gammopathy (1848 mg/dL). A skin biopsy was performed before anakinra administration. Laboratory findings were as follows: monoclonal IgM gammopathy (1656 mg/dL), neutrophilic leukocytosis (WBC, 9790/μL; neutrophils, 77.5%), but normal CRP. Histopathology demonstrated, in addition to the dermis, neutrophilic infiltration within the secretory epithelia of eccrine glands (Figure 2f,g).

### 2.4 Other domestic cases

We contacted attending physicians to reevaluate tissue from previously reported domestic SchS cases.^1^ Out of 12 cases, we could not review tissue in four instances due to either their unavailability from prior biopsies or the extended time since the biopsies were conducted. In the eight cases we were able to assess, we identified neutrophilic epitheliotropism in five cases, while the remaining three cases did not confirm these findings (Table 1).

**TABLE 1:** Presence or absence of neutrophilic epitheliotropism in skin biopsy of patients with Schnitzler syndrome in Japan.

	Findings	Case number in the summary of domestic cases^1^
Cases at Kyoto University Hospital		
Case 1	+	Case 27
Case 2	+	Case 22
Case 3	+	Case 23
Other domestic cases		
Case 4	+	Case 6
Case 5	+	Case 9
Case 6	+	Case 11
Case 7	+	Case 14
Case 8	+	Case 25
Case 9	−	Case 1
Case 10	−	Case 2
Case 11	−	Case 4
Positivity rate	72.7%	

## 3 DISCUSSION

We conducted a histological analysis of three SchS cases that presented at Kyoto University Hospital and eight additional cases in Japan, confirming that neutrophilic epitheliotropism as a diagnostic clue for NUD. In this study, attending physicians were asked to verify findings, and some cases had photographic evidence provided for confirmation. However, not all specimens were thoroughly examined by a specific observer, and the observations were not based on myeloperoxidase (MPO) immunostaining, potentially missing some findings. Nevertheless, in at least three of our cases, we found that findings of neutrophilic epitheliotropism missed by regular histological examination could not be confirmed by MPO staining, suggesting that the visibility of neutrophilic epitheliotropism can vary depending on the section.

The sensitivity of neutrophilic epitheliotropism in NUD was reported as 83.1% by Broekaert et al.^5^ Specifically in SchS, 20 out of 24 specimens (83.3%) from 13 cases were positive. However, it remains unclear whether the findings varied between multiple specimens within the same case or how many cases had positive results individually. Our present study counted the positivity rate on a case base, and although it is possible that some positive findings were missed, the positivity rate was 72.7%. Diagnosing NUD can be challenging when a skin biopsy is performed on a rash clinically resembling urticaria. Hence, we consider the histopathological feature of neutrophil epitheliotropism a valuable diagnostic clue.

Furthermore, monoclonal macroglobulinemia, which is one of the two obligate criteria in Strasbourg criteria,^3^ does not always show high IgM levels in the early stages of the disease, as demonstrated in our study of a collection of Japanese SchS cases.^1^ Wesselmann et al.^8^ reported a case of chronic urticarial‐like rash and elevated WBC counts and CRP levels, but without monoclonal gammopathy. The patient (Case 3) was treated with an anakinra, and her symptoms resolved quickly. Skin biopsies of this patient showed neutrophil epitheliotropism, suggesting an autoinflammatory disease. This report indicates that, even if the essential criteria for the diagnosis of SchS are not met, treatment with IL‐1 inhibitors may still be effective when skin histopathology demonstrates neutrophilic epithelial involvement.

Unfortunately, the exact significance of neutrophilic epitheliotropism remains unclear. In their study, Broekaert et al.^5^ examined normal urticaria with neutrophilic infiltration and lesions of LE with similar infiltration, suggesting that it may not solely indicate neutrophil activation. They speculated that these findings could be related to an autoinflammatory mechanism involving IL‐1. We hope to explore the nature of neutrophils displaying neutrophilic epitheliotropism in SchS.

## FUNDING INFORMATION

This study was supported in part by a research grant from the Ministry of Health, Labour and Welfare, Japan (N.K.) and the Practical Research Project for Rare/Intractable Diseases (JP23ek0109582) from AMED (N.K.).

## CONFLICT OF INTEREST STATEMENT

Naotomo Kambe receives canakinumab free of charge from Novartis for use in an investigator‐initiated clinical trial for Schnitzler syndrome in Japan.
